# Endothelium Derived Nitric Oxide Synthase Negatively Regulates the PDGF-Survivin Pathway during Flow-Dependent Vascular Remodeling

**DOI:** 10.1371/journal.pone.0031495

**Published:** 2012-02-15

**Authors:** Jun Yu, Yuanyuan Zhang, Xinbo Zhang, R. Daniel Rudic, Philip M. Bauer, Dario C. Altieri, William C. Sessa

**Affiliations:** 1 Vascular Biology and Therapeutics Program, Yale University School of Medicine, New Haven, Connecticut, United States of America; 2 Internal Medicine Section of Cardiovascular Medicine, Yale University School of Medicine, New Haven, Connecticut, United States of America; 3 The Wistar Institute Cancer Center, Philadelphia, Pennsylvania, United States of America; Northwestern University, United States of America

## Abstract

Chronic alterations in blood flow initiate structural changes in vessel lumen caliber to normalize shear stress. The loss of endothelial derived nitric oxide synthase (eNOS) in mice promotes abnormal flow dependent vascular remodeling, thus uncoupling mechanotransduction from adaptive vascular remodeling. However, the mechanisms of how the loss of eNOS promotes abnormal remodeling are not known. Here we show that abnormal flow-dependent remodeling in eNOS knockout mice (eNOS (−/−)) is associated with activation of the platelet derived growth factor (PDGF) signaling pathway leading to the induction of the inhibitor of apoptosis, survivin. Interfering with PDGF signaling or survivin function corrects the abnormal remodeling seen in eNOS (−/−) mice. Moreover, nitric oxide (NO) negatively regulates PDGF driven survivin expression and cellular proliferation in cultured vascular smooth muscle cells. Collectively, our data suggests that eNOS negatively regulates the PDGF-survivin axis to maintain proportional flow-dependent luminal remodeling and vascular quiescence.

## Introduction

Physiological adaptive remodeling occurs in response to changes in blood flow [1]. In conduit vessels, increases in blood flow will increase lumen diameter, while decreases in blood flow will reduce lumen diameter in order to normalize shear stresses associated with changes in blood flow. These changes in lumen diameter are secondary to structural changes triggered by precise regulation of apoptosis versus proliferation, metalloproteinase activation, coordinated cell migration and organization to change lumen diameter while keeping wall thickness constant [2], [3], [4], [5].

The molecular mechanisms necessary for adaptive and injury evoked remodeling are beginning to be appreciated. In a surgical model, reducing blood flow by 30–40% reduces vessel diameter without changing wall thickness by an endothelium dependent mechanism. Denudation of the endothelium prevents flow mediated inward remodeling demonstrating that the endothelium senses and transmits the changes in shear stress into biochemical signals that coordinate structural changes in the vessel wall [4]. Previous work has shown that endothelial nitric oxide synthase (eNOS) is critical for flow-dependent adaptive remodeling, injury evoked inward remodeling and ischemia induced shear stress dependent collateral artery remodeling [6], [7], [8], [9]. Reducing blood flow in mice deficient in eNOS prevents flow-dependent inward luminal remodeling and promotes vascular smooth muscle proliferation leading to an increase in wall thickness [6], [10]. Thus, the loss of eNOS derived nitric oxide (NO) uncouples hemodynamic changes from adaptive physiological remodeling.

The mechanisms by which the loss of eNOS regulates flow dependent remodeling are unknown. Here we show that the loss of eNOS increases platelet derived growth factor (PDGF) expression with the concomitant induction of the anti-apoptotic protein, survivin. Interference with either PDGF signaling or antagonizing the actions of survivin reverses the abnormal remodeling in eNOS (−/−) mice. In addition, exogenous NO reduces PDGF mediated proliferation of vascular smooth muscle cells and survivin induction, *in vitro*. Thus, eNOS negatively regulates the PDGF-survivin axis to govern adaptive flow dependent remodeling.

## Results

### Congenic eNOS (−/−) mice have impaired vascular remodeling and exhibit media hyperplasia

**Figure 1:**
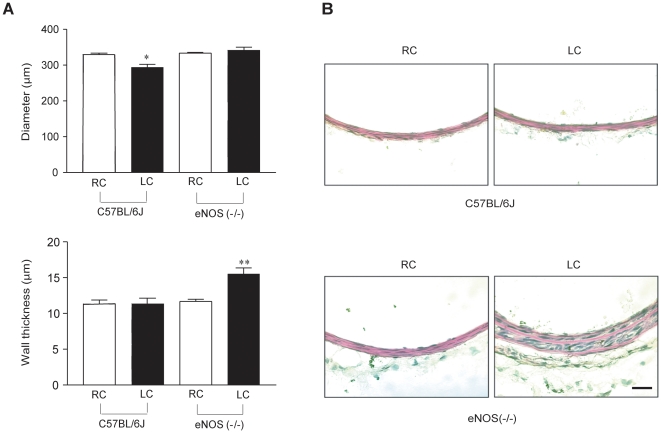
Impaired vascular remodeling in congenic eNOS knockout mice. (A) Morphometric analysis showing reductions in lumen diameter of LC in C57BL/6J mice with no change in wall thickness in response to a remodeling stimulus, yet no change in lumen diameter but an increase in wall thickness in LC of eNOS (−/−) mice. (B) Hematoxylin and eosin staining showing RC and remodeled LC from a C57BL/6J mouse (upper panel) and RC and remodeled LC from an eNOS (−/−) mouse (lower panel). Scale bar represents 25 µm. Values are mean ± SEM; * P<0.05, ** P<0.01 with one way ANOVA with Bonferroni posttest; n = 5 for each group of mice.

In our previous study, ligation of the left external carotid artery (LEC) in F2 generation eNOS (−/−) mice triggered impaired flow-dependent remodeling in the left common carotid artery (LC) and paradoxically increased wall thickness via medial cell proliferation [6]. As seen in Figure 1A, ligation of the LEC for 7 days in C57BL/6J mice reduces lumen diameter by approximately 12% in the LC, without changing wall thickness. In contrast, ligation of LEC in congenic eNOS (−/−) mice did not change LC lumen diameters but markedly increased wall thickness (by 33%) consistent with our previous data in F2 mice showing that eNOS was critical for proportional remodeling of lumen and wall thickness. As seen in Figure 1B, the remodeled LC of C57BL/6J mice is indistinguishable from the contralateral right carotid artery (RC) or the RC of eNOS (−/−) mice, while the remodeled LC of eNOS (−/−) mice shows obvious increases in wall thickness.

### Chronic decreases in blood flow in LC of eNOS (−/−) mice induce PDGF-AA/BB and survivin gene expression

**Figure 2:**
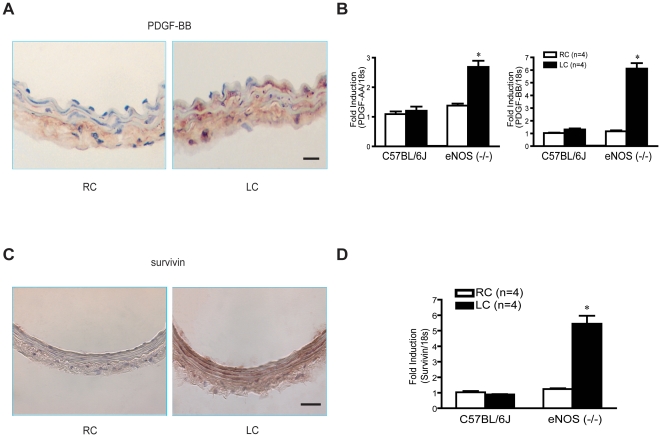
PDGF-AA, PDGF-BB and survivin levels are increased in eNOS (−/−) mice in response to remodeling stimulus. (A), Immunohistochemistry of PDGF-BB protein shows that it is present in adventitia of RC but is increased in all layers of LC post-ligation. Scale bar, 10 µm. (B), Quantitative RT-PCR of PDGF-AA and BB shows there was no change of PDGF mRNA level in wild type mice after LEC ligation. However, PDGF-AA and BB mRNA are all elevated in ligated LC compare to contralateral RC in eNOS (−/−) mice, 18 s was used as internal control. (C), Survivin immunohistochemistry shows strong staining throughout whole vessel wall of LC, and there is little or no staining in RC. Scale bar equals to 10 µm. (D), RT-PCR shows equal expression of survivin mRNA of contralateral RC and ligated LC in wild type mice. However, survivin was upregulated in ligated LC of eNOS (−/−) mice. 18 s was used as internal control.

The PDGF isoforms (AA and BB) are well characterized, potent chemotactic and mitogenic growth factors for vascular smooth muscle cells. To examine if PDGF levels were elevated in abnormally remodeled eNOS (−/−) mice, we performed immunohistochemistry and quantitative RT-PCR. Figure 2A shows greater immunoreactive PDGF-BB protein levels in all layers of abnormally remodeled LC of eNOS (−/−) mice compared to RC. The mRNA for PDGF-AA, PDGF-BB, VEGF-A, FGF2 and TGFb1 is present in RC and remodeled LC of C57BL/6J detected by quantitative RT-PCR. However, the expressions of both isoforms of PDGFs, but not other growth factors were enhanced in abnormally remodeled LC of eNOS (−/−) mice compared to the paired RC from the same mouse 7 days post-surgery (Fig. 2B, Fig. S1). These findings suggest that PDGF may play an important role in abnormal remodeling and medial proliferation in eNOS (−/−) mice.

Previous data from our group has discovered that PDGF can induce the expression of the inhibitor of apoptosis, survivin, in vascular smooth muscle promoting medial cell proliferation after vascular injury [11]. Thus, we examined survivin expression in flow-remodeled arteries. Immunoreactive survivin protein (Fig. 2C) and mRNA levels (Fig. 2D) were elevated in remodeled LC of eNOS (−/−) mice compared to the contralateral vessel. Low levels of survivin immunoreactivity and mRNA were found in C57BL/6J mice or non-remodeled RC of eNOS (−/−) mice. Given that survivin is an inhibitor of apoptosis, we examined the apoptosis via TUNEL in remodeled LC of C57Bl/6J and eNOS (−/−) mice. Apoptosis was detected in the LC in both strains of mice, but no differences were found in eNOS (−/−) versus C57BL/6J mice (Fig. S2). These data suggest that the abnormal LC remodeling in eNOS (−/−) mice was not due to the direct anti-apoptosis effect of survivin, and perhaps survivin control of cell cycles contributes to the actions of survivin during remodeling.

### PDGF-BB receptor tyrosine kinase inhibitor corrects pathological remodeling in eNOS knockout mice by inhibiting vascular smooth muscle cell proliferation and reducing survivin levels

**Figure 3:**
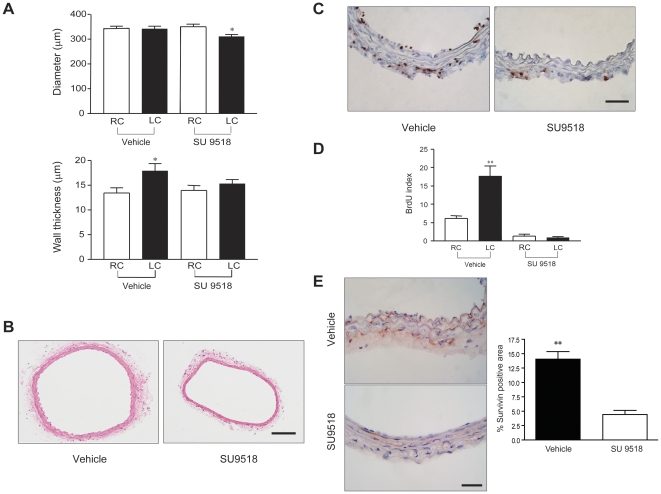
SU9518 decreases cell proliferation and restore normal remodeling in eNOS (−/−) mice. (A), Lumen diameters (upper panel) of LC in SU9518 treated eNOS (−/−) mice were reduced in response to a remodeling stimulus but not in LC of vehicle treated eNOS (−/−) mice. Wall thickness (lower panel) of vehicle treated LC was significantly increased and, SU9518 restored the normal wall remodeling in LC. Values are mean ± SEM; n = 7 for each group of mice; * P<0.05, ** P<0.001 with one way ANOVA with Bonferroni posttest. (B) Representative hematoxylin and eosin stained LC cross sections of vehicle or SU9518 treated mice. Inward remodeling can be seen in SU9518 treated LC. Scale bar represents 100 µm. (C), Immunostaining shows BrdU positive cells were detected in all layers of ligated LC in vehicle treated mice (left panel), which blocked by SU9518 treatment as shown on the right panel. Scale bar represents 25 µm. (D), Quantitative BrdU index shows cell proliferation was significantly increased in vehicle treated LCs of eNOS (−/−) mice, but not in vessels from SU9518 treated eNOS (−/−) mice. (E) PDGF-BB receptor tyrosine kinase inhibitor decreases immunoreactive survivin levels in LC of eNOS (−/−) mice, compared to vehicle treated eNOS (−/−) mice (left panel). Right panel shows the quantification of percentage of suvivin positive staining in total vessel area. Values are mean ± SEM; n = 5. * P<0.05, ** P<0.001 with one way ANOVA with Bonferroni posttest.

To examine if upregulation of PDGF contributed to the abnormal remodeling of eNOS (−/−) mice, we treated eNOS (−/−) mice (daily from one day before surgery to 7 days post-surgery) with SU9518, an orally active inhibitor of PDGF receptor tyrosine kinase, that has been shown to selectively inhibit PDGF mediated smooth muscle cell migration and proliferation in vivo [12]. SU9518 competitively inhibits the tyrosine kinase activity of the PDGF receptors (α and β) and does not influence EGF or FGF receptor tyrosine kinase signaling (Table 1). Moreover, this compound has good oral bioavailability and blocks phosphorylation of the PDGF receptors in carotid arteries isolated from rats treated in vivo. As shown in Figure 3A, reducing blood flow in eNOS (−/−) mice did not result in inward remodeling of LC and triggered an increase in wall thickness (compared to contralateral RC). However, treatment with SU9518 promoted inward remodeling of LCs of eNOS (−/−) mice and reduced wall thickness back to control levels (Fig. 3A and histologically seen in Fig. 3B). To further test the mechanism of how SU9518 restored normal remodeling in eNOS (−/−) mice, *in vivo* cell proliferation was assessed via BrdU incorporation. Immunohistochemistry of BrdU showed a marked reduction in BrdU positive cells throughout endothelium, media and adventitia layer of the vessels in SU9518 treated LC compare to vehicle treated LC (Fig. 3C) and the BrdU labeling index showed a significant decreased in the ratio of proliferating cells/total cells in the vessel wall (Fig. 3D). Finally, we examined immunoreactive survivin protein levels in eNOS (−/−) mice treated with SU9518. As seen in Figure 3E, vehicle treated eNOS (−/−) mice had ample survivin labeling in abnormally remodeled LC, whereas treatment with SU9518 reduced these levels to background levels. These data demonstrate that inhibition of PDGF receptor signaling prevents pathological outward remodeling in eNOS (−/−) mice by decreasing cell proliferation and survivin levels.

**Table 1:** SU9518 selectively inhibits PDGFR.

Enzyme	IC_50_ (µM) mean±sem (n)
PDGFR	<0.005±0.001 (3)
VEGFR2	3.3±2.6 (6)
FGFR1	2.5±0.5 (3)
Src	5.8±7.5 (8)
FAK	40.5 (1)
EGFR	>100 (2)

### Adenoviral gene delivery of dominant negative, phosphorylation-defective survivin (T34A) reduces abnormal remodeling in eNOS knockout mice

**Figure 4:**
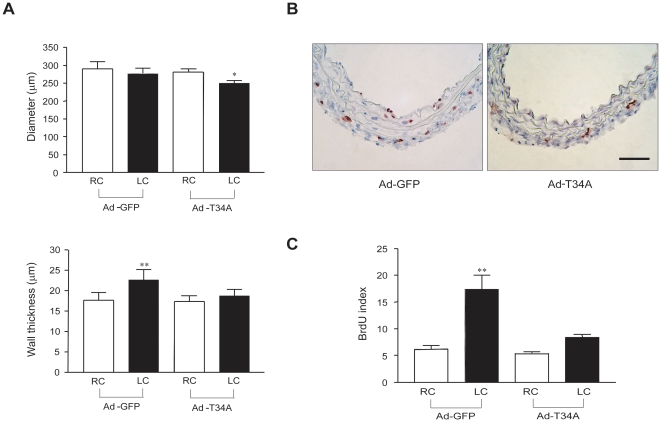
Ad-T34A survivin transduction blocks cell proliferation and restores normal remodeling in eNOS (−/−) mice. (A) LCs were transduced with Ad-GFP or Ad-T34A survivin at the time of LEC ligation. Lumen diameter and wall thickness were calculated from 10 sections of contralateral RC and infected LC of eNOS (−/−) mice. Upper panel shows lumen diameter of Ad-T34A transduced LC was significantly decreased compared to RC. Ad-T34A transduction also blocks the wall thickening in transduced LC (lower panel). (B), Ad T34A suvivin reduces the number of BrdU positive cells in LC. (C), BrdU labeling index shows significant increase of BrdU positive cell/total nuclei ratio in Ad-GFP transduced LC compare to RC in eNOS (−/−) mice. This activation of cell proliferation was blocked by Ad-T34A transduction. Values are mean ± SEM; * P<0.05, ** P<0.01, and † P<0.001 with one way ANOVA with Bonferroni posttest; n = 5 for each group of mice. Scale bar equals to 25 µm.

To examine if the upregulation of survivin was coincidental or causal in the abnormal flow-dependent remodeling found in eNOS (−/−) mice, we used adenoviral (Ad) gene transfer of either Ad-GFP or Ad-T34A survivin. The T34A mutation in survivin prevents phosphorylation of the endogenous survivin by the mitotic kinase p34^cdc^ - cyclin B and results in reduced cell growth and apoptosis [11], [13], [14]. Viruses were applied to the adventitial side of the vessel wall immediately after LEC ligation using pluronic gel as a vehicle for gene delivery and vessel morphometry measured 7 days later. As document in previous studies, pluronic acid delivery of Ad-GFP penetrates all layers of the vessel wall, with a gradient from adventitia to intima [11], [15]. As seen in Figure 4A, transduction of LC from eNOS (−/−) mice with Ad-T34A survivin, but not Ad-GFP, significantly decreased LC lumen diameter (upper panel), and reduced the medial thickening (lower panel) comparable to that observed in WT mice (see Fig. 1). Moreover, the incorporation of BrdU (Fig. 4B) was reduced in Ad-T34A transduced arteries as quantified as BrdU positive cells/total cells per section (Fig. 4C), throughout the vessel wall compared to Ad-GFP transduced vessels. These data suggest that interference with survivin function can partially correct the impaired vascular remodeling seen in eNOS (−/−) mice.

### Nitric oxide blocks PDGF-induced cell proliferation and survivin expression in vitro

**Figure 5:**
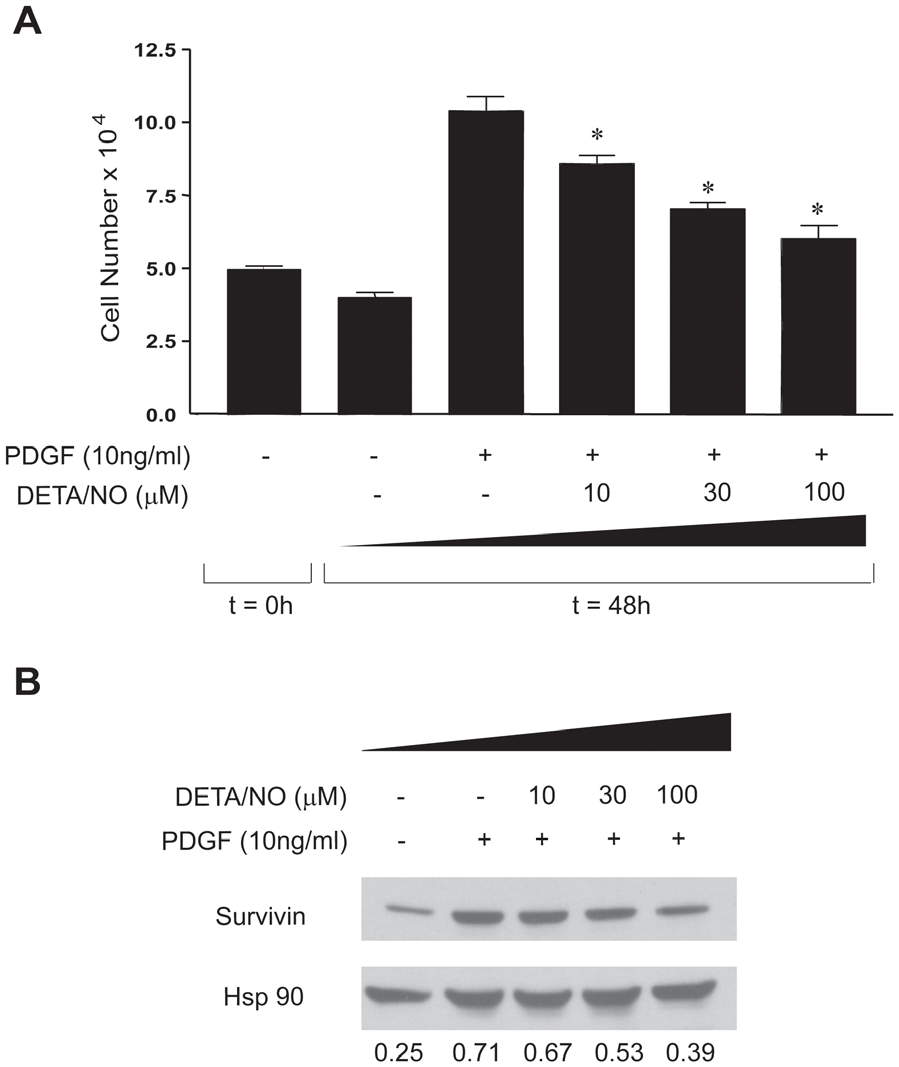
NO donor dose dependently decreases cell number induced by PDGF-BB and blocks survivin expression and *in vitro*. (A), Rat SMC were treated or not with PDGF (10 ng/ml), in the absence or presence of the NO donor, DETA/NO (10, 30, and 100 µM) and cell number quantified after 48 h. (B), NO reduces PDGF induced survivin levels. VSM were treated with PDGF (10 ng/ml) with or withoutt DETA/NONO (10, 30 and 100 µM) and the levels of survivin assessed by Western blotting. DETA/NO dose dependently decreased survivin levels relative to Hsp90 (a protein loading control). Densitometric values of the ratio of survivin to Hsp90 are shown below the blots. * P<0.05, ** P<0.01 with one way ANOVA with Bonferroni posttest.

Our *in vivo* data implies that the loss of endogenous eNOS derived NO permits activation of the PDGF-survivin axis thereby promoting abnormal vascular smooth muscle cells (VSMC) proliferation and aberrant vascular remodeling. To directly examine if NO could regulate PDGF stimulated signaling leading to the induction of survivin, we initially examined the well-characterized effects of NO on PDGF driven proliferation of cultured VSMC. As seen in Figure 5A, PDGF (10 ng/ml) induced a doubling in cell number after 48 hrs, while the addition of an NO donor, DETA/NO, dose-dependently reduced PDGF stimulated VSMC proliferation. Interestingly PDGF induces the expression of survivin in VSMC (Fig. 5B), an effect dose-dependently antagonized by the addition of DETA/NO. These data show that NO can negatively regulate PDGF mediated survivin induction and support our in vivo findings demonstrating that the loss of endothelial derived NO promotes this pathway leading to abnormal vascular remodeling.

## Discussion

Here we show that the abnormal vascular remodeling triggered by the loss of endothelium-derived NO is linked to activation of the PDGF-survivin pathway. Antagonism of either PDGF receptor tyrosine kinase activity with SU9815 or endogenous survivin with adenoviral delivery of a phosphorylation defective mutant of survivin prevents abnormal carotid arterial remodeling in eNOS (−/−) mice thereby normalizing flow-dependent luminal remodeling to that typically observed in wild-type mice. These studies support the concept that endogenous endothelium-derived NO maintains vascular homeostasis by negatively regulating the PDGF-survivin balance and implies that endothelial dysfunction, characterized by a loss of NO bioactivity, may promote the early phases of abnormal vascular remodeling through this mechanism. This concept is additionally supported by previous data showing that in two distinct vascular injury models characterized by endothelial dysfunction, the PDGF-survivin axis regulates medial thickening [11].

Although it has been appreciated for 20 years that the endothelium regulates the interface between hemodynamics and vascular structure, however, the precise molecular mechanisms in vivo are less well understood. Physiologically, chronic flow alterations can regulate proportional vascular remodeling either to increase or decrease lumen diameter. The change in lumen diameter is necessary to normalize shear stresses brought about by the flow changes. Using genetically altered mice, eNOS was the first gene shown to be critical for post-natal, flow-dependent remodeling [6]. It is important to emphasize that merely changing blood flow is a non-inflammatory, hemodynamic remodeling stimulus different from severe ligation models that stimulate massive neointima formation and luminal narrowing [16], [17]. Upon reducing blood flow in eNOS (−/−) mice, the endothelium cannot regulate shear stress, thus increasing the proliferation of vascular smooth muscle, increasing wall thickness to normalize wall strain. Here, we show that a likely mediator of VSMC proliferation in the absence of eNOS is PDGF. PDGF levels are elevated in the absence of eNOS in abnormally remodeled vessels, but not the contralateral right carotid artery, suggesting that the inability to regulate shear stress stimulates PDGF synthesis. We didn't find much change of other growth factors and their receptor levels in the remodeled arteries. But, this could be limited by the specific time point we studied. It is worth doing a time course study in the future. Moreover, antagonism of PDGF receptors with SU9518, prevents dysregulated luminal remodeling in eNOS (−/−) mice, inhibits abnormal VSMC proliferation and corrects the defect in wall thickness. It can be argued that this inhibitor may block other growth factor regulated pathways leading to abnormal remodeling, and we cannot rule this out. However, the presence of PDGF isoforms and cellular hyperproliferation in abnormally remodeled vessels from eNOS (−/−) mice, the well-established actions of NO in antagonizing PDGF induced VSMC proliferation and migration [18], [19], [20], [21], in conjunction with the data showing SU9518 blocks BrdU incorporation and abnormal remodeling in eNOS (−/−) strongly supports PDGF as a stimulus for abnormal remodeling.

PDGF can activate many signaling pathways that promote abnormal vascular remodeling. Previous work has shown that PDGF and angiotensin II can induce survivin gene and protein expression in VSMC in vitro and in vivo [11], [22]. Survivin was discovered as a novel transcript that was expressed in a variety of cancer cells and elevated survivin levels are associated with greater mortality in a variety of human cancers [23], [24], [25]. It is structurally and functionally an inhibitor of apoptosis (IAP) due its BIR domains and functional data showing that survivin expression inhibits apoptosis in many cell types. Recently, work from several groups have shown that certain growth or survival factors such as VEGF or angiopoietin-1 can also induce survivin expression and reduce the apoptotic threshold to diverse apoptogens in differentiated, primary cultures of endothelial cells [26], [27], [28]. In the context of most cells, the induction of survivin affords an anti-apoptotic environment during G2/M progression of the cell cycle [13]. Clearly, antagonism of survivin function with the phospho-mutant T34A survivin, increases the apoptotic threshold and reduces cellular proliferation [11], [13], [14]. In previous work, we have shown in two distinct models of vascular injury (associated with elevated levels of PDGF) that survivin levels are induced in the expanding neointima. Antagonism of survivin function using gene delivery of adenovirus encoding T34A survivin reduced neointimal expansion, BrdU incorporation and promoted apoptosis [11]. Here, we show that eNOS derived NO tonically inhibits the PDGF-survivin axis in a non-injurious, non-inflammtory, flow reducing model of vascular remodeling. Thus, in the absence of gross endothelial loss and activation of the coagulation system typically seen in injury paradigms, we show the loss of eNOS is associated with elevation of the PDGF-survivin pathway after a flow reducing remodeling stimulus. Neutralization of either PDGF signaling or survivin function improves the abnormal remodeling seen in eNOS (−/−) mice. In addition to this clear evidence in vivo, mechanistically exogenous NO inhibits PDGF stimulated survivin induction and cellular proliferation in cultured VSMC. The data showing that a reduction in endogenous NO derived from endothelium promotes survivin upregulation, and the exogenous NO to reduces PDGF stimulated survivin levels extends the observation that high concentrations of NO donor drugs decrease survivin expression in a variety of cancer cells [29]. Thus, we surmise that the loss of NO bioactivity, either via injury, secondary to endothelial dysfunction or genetically as in this study, has the propensity to promote an anti-apoptotic, pro-proliferative environment conducive to neointima formation or abnormal remodeling.

## Materials and Methods

### Left external carotid artery ligation in mice

All animal studies were approved by the institutional animal care and use committees of Yale University. Eight to ten weeks old male congenic (backcrossed at least nine generations to the C57Bl/6J background) eNOS (−/−) or C57BL/6J mice (Jackson Laboratories, Bar Harbor, ME) were anesthetized with ketamine/xylazine (79.5 mg/kg ketamine, 9.1 mg/kg; xylazine). Left external carotid artery ligation and adenoviral gene delivery were performed essentially as previously described [6], [30]. Briefly, the left external carotid artery was ligated from its origin with 7-0 nylon suture (USSC, Norwalk, CT). In some experiments, eNOS (−/−) mice were treated with vehicle [1.0% carboxymethylcellulose (CMC) in saline] or SU9518 (3.0%; w/v in CMC vehicle), an inhbitor of PDGF receptor tyrosine kinase [12] daily by gavage from one day before surgery to sacrifice day. In additional experiments, adenoviruses encoding phosphorylation-defective survivin (Ad-T34A) or green fluorescent protein (Ad-GFP, 3×10^8^ PFU) were delivered by painting the adventitial side of the carotid artery with 50 µl of adenovirus/30% Pluronic-127 gel (Sigma, St. Louis, MO) mixture immediately after ligation [11], [31]. All animals were injected with 5-bromo-2′deoxyuridine (BrdU) subcutaneously (25 mg/kg) 3 days before sacrifice daily and intraperitoneally (30 mg/kg) 12 hr and 24 hr before death to label proliferating cells. BrdU index was the percentage of BrdU positive cells of total nuclei in endothelium, media and adventitia. Carotid arteries were collected at day 7 post-surgery for morphometric analysis, immunohistochemistry or RNA isolation.

### Histology and immunohistochemistry

At sacrifice, perfusion fixed (4% paraformaldehyde in PBS, PH 7.4) carotid arteries were taken and embedded in O.C.T (Tissue-Tek, Elkhart, IN). Cryosections (5 µm) of arteries were obtained for hematoxylin/eosin, elastic staining and immunohistochemistry. For immunohistochemistry, artery sections were quenched for endogenous peroxidase, blocked by 10% goat serum and incubated with anti-PDGF BB (R & D systems, Minneapolis, MN), anti-survivin or anti-BrdU (Pharmingen). Bound primary antibodies were detected using avidin-biotin-peroxidase (NovaRed™ peroxidase substrate kit, Vector Laboratories, Burlingame, CA).

### Terminal Deoxynucleotidyl Transferase End Labeling

Apoptotic cells in vascular wall were labeled by TUNEL (TdT-mediated dUTP nick-end labeling) using the *in situ* cell death detection kit TMR-red (Roche Diagnostics) according to the manufacturer's protocol. Briefly, dewax paraffin embedded sections in xylene and rehydrate in graded ethanol series to water. Heat slides in 10 mM sodium citrate buffer (pH 6.0) at 95–100°C for 10 minutes and then incubate slides in permeabilisation solution (0.1% Triton X-100, 0.1% sodium citrate in PBS buffer) for 2 min on ice. After dialyzed extensively against PBS buffer for 30 min at room temperature, the slides were incubated with 50 µl TUNEL reaction mixture covered with Para film pieces for 60 min at 37°C in a humidified atmosphere in the dark. To identify cell types undergoing apoptosis, double staining was performed by combining TUNEL and immunohistochemistry for α-SMC actin (smooth muscle cells). TUNEL staining sections were then viewed using inverted fluorescent microscope and take photography. Only TUNEL-positive cells that colocalized with DAPI-stained nuclei were counted as positive and three sections of each mouse were quantified to detect the apoptotic cell number.

### Morphometric analysis

Elastic staining was done on 10 cross-sections from 5 carotid arteries for each group as previously described. Cross-section images were collected using a Zeiss microscope and on line CCD camera (DAGE-MTI, Michigan City, Indiana). The circumference of internal elastic lamina (IEL) was measured and artery diameter was calculated. The distance from IEL to external elastic lamina (EEL) in four locations of each section was measured by image analysis software (Scion Image, Frederick, Maryland). The average was taken as wall thickness.

### RT-PCR

Total RNA of single carotid artery was isolated by using phenol/chloroform. Quantitative RT-PCR was then performed using 200 ng RNA respectively and primers specific for murine PDGF-AA, PDGF-BB, PDGFBR, survivin, TGFb1, TGFBR2, FGF2, FGFR1–4, ANG1, ANG2, TIE1, TIE2, VEGFA, VEGFR1, VEGFR2, and, 18 s was used as an internal control.

### Effects of NO on PDGF-induced smooth muscle proliferation and survivin expression

A10 rat aortic VSMC were cultured in high glucose Dulbecco's modified Eagle's medium containing 10% (v/v) fetal bovine serum, penicillin, streptomycin, and L-glutamine. For experiments VSMC were plated at a density of 5,000 cells/cm^2^ into six well cell culture plates and incubated overnight in normal growth medium. The next day the cells were washed 2 times with PBS and 2 ml of serum free DMEM was added to each well. After 24 hours 15 ng/ml PDGF was added to each well with or without varying concentrations of DETANO/NOate ((Z)-1-[N-(2-aminoethyl)-N-(2-aminoethyl)-amino]-diazen-1-ium-1,2-diolate). The cells were incubated for a further 48 hours and the cells were trypsinized and counted in a Coulter particle counter. To examine survivin expression, VSMC were plated at approximately 50% confluency in 10 cm culture dishes and incubated overnight. The next day the cells were washed 2 times with PBS and 2 ml of serum free DMEM was added to each well. After 24 hours, PDGF was added to each well with or without varying concentrations of DETANO/NOate and incubated for a further 24 hours. The cells were then washed 2 times with ice cold PBS and lysed on ice in 50 mM Tris HCL, pH 7.5, 1% Nonidet P-40 (v/v), 10 mM NaF, 1 mM vanadate, 1 mM pefabloc, 10 mg/ml leupeptin, and lysates were transferred to microcentrifuge tubes and rotated for 45 min at 4°C. Insoluble material was removed by centrifugation at 12,000 g for 10 min at 4°C and 20 µg of protein from cell lysates were analyzed by Western Blot analysis for survivin or hsp90.

## Supporting Information

Figure S1 **Growth factor and receptor expression in ligated LC in C57Bl/6J and eNOS (−/−) mice.** Quantitative RT-PCR was performed using single remodeled LC and contralateral RC from C57BL/6J and eNOS (−/−) mice. There was no change of mRNA level of multiple growth factors and their receptors in LC of wild type mice 7 days after LEC ligation. However, there was a trend of induction (2-fold but not significant compared to WT) of FGF2, FGFR2, TGFbR2, Ang2 and Tie1 expression in LC compared to RC in eNOS (−/−) mice. N = 3, one way ANOVA was used for statistical analysis.
(TIF)

Figure S2 **Loss of eNOS does not alter cell apoptosis in response to flow alteration in vivo.** (A) Representative images of TUNEL stained remodeled LC from C57Bl/6J and eNOS (−/−) mice 7 days after LEC ligation. TUNEL label in red, SMA staining in green. (B) Quantification of percentage of TUNEL positive cells in total vascular wall cells. N = 3 animal, student T-test was used for statistical analysis.
(TIF)
