# Supplementary figures and images for: Endothelium Derived Nitric Oxide Synthase Negatively Regulates the PDGF-Survivin Pathway during Flow-Dependent Vascular Remodeling
Source: PLoS One. 2012 Feb 15;7(2):e31495. doi: 10.1371/journal.pone.0031495 (PMC3280303; doi:10.1371/journal.pone.0031495)

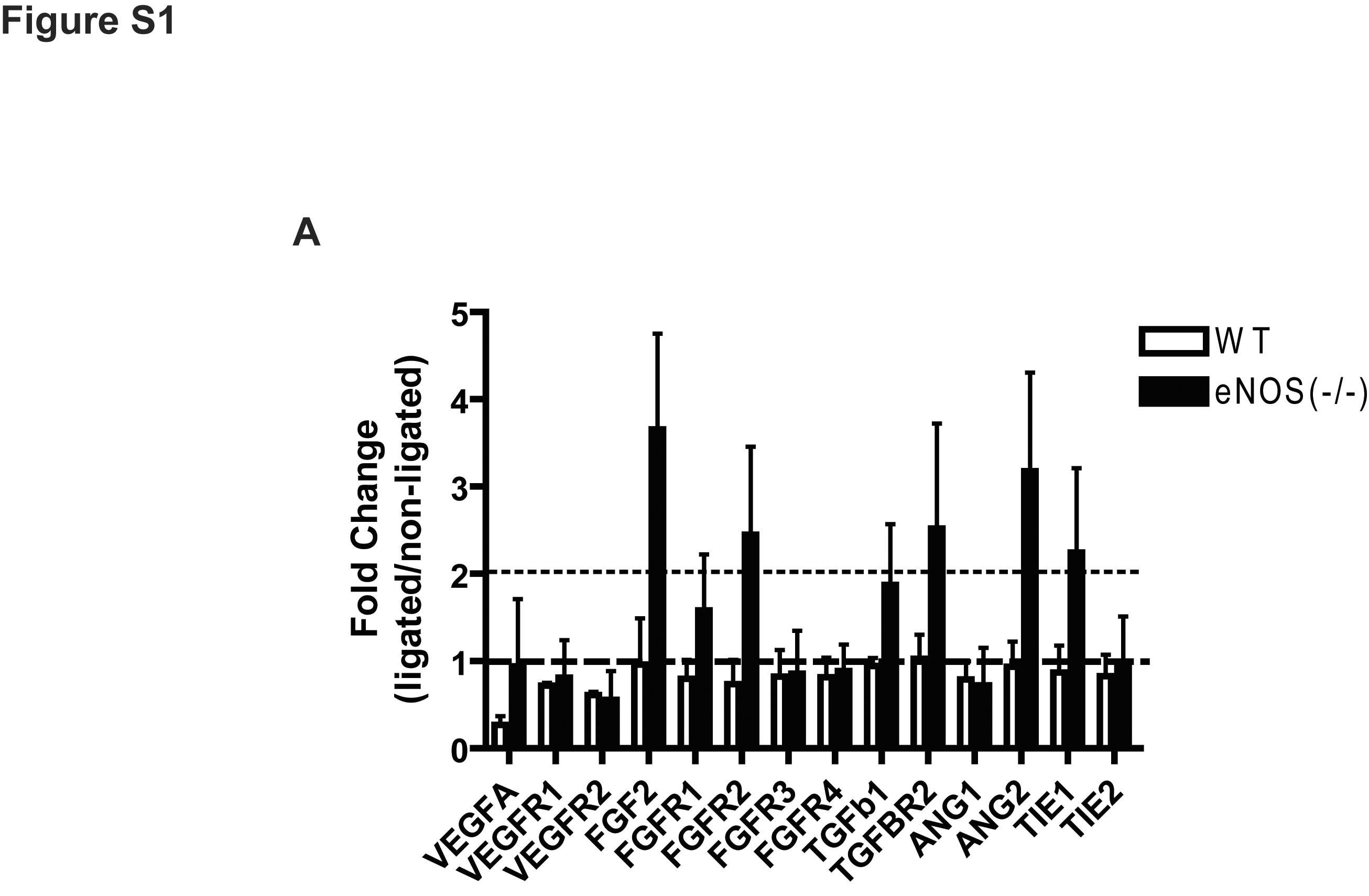

Supplement: Figure S1 — Growth factor and receptor expression in ligated LC in C57Bl/6J and eNOS (−/−) mice. Quantitative RT-PCR was performed using single remodeled LC and contralateral RC from C57BL/6J and eNOS (−/−) mice. There was no change of mRNA level of multiple growth factors and their receptors in LC of wild type mice 7 days after LEC ligation. However, there was a trend of induction (2-fold but not significant compared to WT) of FGF2, FGFR2, TGFbR2, Ang2 and Tie1 expression in LC compared to RC in eNOS (−/−) mice. N = 3, one way ANOVA was used for statistical analysis. (TIF) [file pone.0031495.s001.tif]

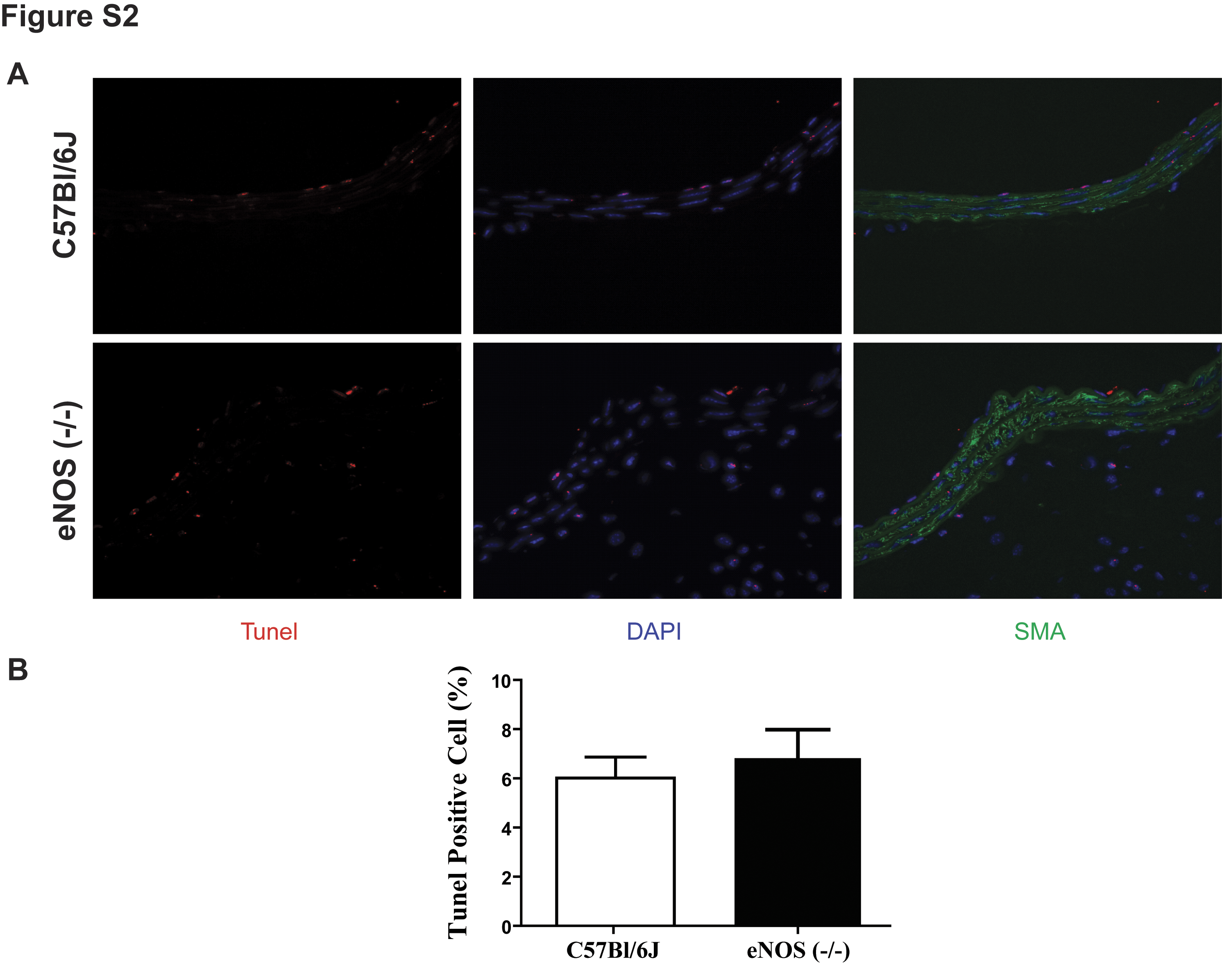

Supplement: Figure S2 — Loss of eNOS does not alter cell apoptosis in response to flow alteration in vivo. (A) Representative images of TUNEL stained remodeled LC from C57Bl/6J and eNOS (−/−) mice 7 days after LEC ligation. TUNEL label in red, SMA staining in green. (B) Quantification of percentage of TUNEL positive cells in total vascular wall cells. N = 3 animal, student T-test was used for statistical analysis. (TIF) [file pone.0031495.s002.tif]
